# Public health practitioner perspectives on dealing with measles outbreaks if high anti-vaccination sentiment is present

**DOI:** 10.1186/s12889-021-10604-3

**Published:** 2021-04-09

**Authors:** Penelope Robinson, Kerrie Wiley, Chris Degeling

**Affiliations:** 1 Sydney School of Public Health, Faculty of Medicine & Health, University of Sydney, Edward Ford Building, Sydney, NSW Australia; 2 Australian Centre for Health Engagement, Evidence & Values (ACHEEV), School of Health and Society, Faculty of the Arts, Social Sciences & Humanities, University of Wollongong, Northfields Ave, Wollongong, NSW Australia

**Keywords:** Australia, Vaccination, Immunisation, Health policy, Communicable disease, Expert opinions, Delphi study, Measles, Disease outbreak

## Abstract

**Background:**

Communities with low vaccination rates are at greater risk during outbreaks of vaccine preventable diseases. Most Australian parents support vaccines, but some refuse and are often judged harshly by their community, especially during an outbreak. We sought the perspectives of Australian public health experts on the key issues faced when managing a measles outbreak in an area with high anti-vaccination sentiment.

**Methods:**

A measles outbreak scenario formed the basis of a 3-round modified Delphi process to identify key practitioner concerns in relation to parents/carers who don’t follow the recommended vaccination schedule. We surveyed a range of professionals in the field: policymakers, infectious disease experts, immunisation program staff, and others involved in delivering childhood vaccinations, to identify key priorities when responding to an outbreak in a community with low vaccination coverage.

**Results:**

Findings indicate that responses to measles outbreaks in communities with high anti-vaccination sentiment are motivated by concerns about the potential for a much larger outbreak event. The highest operational priority is to isolate infected children. The two most highly ranked practical issues are mistrust from non-vaccinating members of the local region and combatting misinformation about vaccines. Trying to change minds of such individuals is not a priority during an outbreak, nor is vaccinating their children. Using media and social media to provide information about the outbreak and measures the public can take to limit the spread of the disease was a focus.

**Conclusions:**

Our findings provide a deeper understanding of the challenges faced during an outbreak and priorities for communicating with communities where there is a high level of anti-vaccination sentiment. In the context of a global pandemic, the results of this study also have implications for managing public health responses to community transmission of SARS-CoV-2, as COVID-19 vaccines becomes widely available.

**Supplementary Information:**

The online version contains supplementary material available at 10.1186/s12889-021-10604-3.

## Background

The COVID-19 pandemic has highlighted that public health responses to an infectious disease outbreak are multi-faceted. The responsibility for instigating and driving the response lies with public health authorities. However, the success of the response extends beyond the actions of public health professionals alone. The actions of the public, the responses and policies of institutions such as schools and preschools, the framing and reporting of the situation by the media, and politicisation of the issue, all play a role in the course and management of the outbreak. Illumination of the needs and priorities of the public health professionals responsible for managing an outbreak is vital to inform measures which might ensure the most helpful responses from the other implicated stakeholders. Particularly in a scenario where a preventive measure, such as a vaccine, is available, but not necessarily embraced by the affected population.

Against this background, there has been a significant global resurgence of measles in recent years, with a three-fold increase in cases reported to the World Health Organization (WHO) in the first 6 months of 2019 compared with the same time period in 2018, including serious outbreaks in Western Pacific countries [1]. In 2014 the WHO declared measles eliminated in all states and territories of Australia [2] however 2019 saw 285 confirmed cases in Australia, the highest number since elimination was declared [1]. This is consistent with Australian modelling which shows that even within a setting where elimination is believed to have been achieved, the risk of large outbreaks may remain due variation in vaccination coverage within geographic regions [3]. Measles cases are monitored nationally, and outbreaks are controlled and managed at a local level by Public Health Units according to the relevant State Health Department guidelines. Australia has a national immunisation register (AIR) administered by Medicare Australia which records all vaccinations for adults, adolescents and children in Australia. Public Health Units have access to the register and work in collaboration with local GPs and schools to identify those who are most at risk during an outbreak. The public health response generally involves laboratory confirmation of all suspected cases, isolation of suspected cases from school, childcare or work, and contact tracing. Contact management includes ascertainment of immunity and recommendation of either immunisation or immunoglobulin to contacts where indicated [4]. Contacts deemed susceptible are excluded from school, Early Childhood Education and Care and healthcare settings until 14 days after the first appearance of the rash in the last case. Other response measures include communication campaigns through local media and in some cases setting up of mobile immunisation clinics, for example, at affected schools [5–8].

National immunisation coverage rates for 1, 2 and 5 year-old children in Australia are high compared to the rest of the world, but have yet to reach the aspirational target of 95% [9]. Mandates for childhood vaccines have been introduced in recent years in order to begin to bridge this gap by addressing vaccine refusal. At the National level, in 2016 the Australian government introduced new immunisation mandates, requiring children to be fully immunised to be eligible to receive certain government payments, such as childcare benefits and removing avenues for conscientious objection. More recently, four states (New South Wales, Queensland, Victoria and Western Australia) have introduced “No Jab No Play” policies which require children to be fully immunised in order to be enrolled in a childcare service [10].

In order to provide a more nuanced and detailed understanding of the practical public health implications of vaccine refusal, and to hear the voices of people who work directly with the public during a disease outbreak, in 2019 we conducted a three-round modified online Delphi survey with Australian health professionals and immunisation experts who are responsible for controlling communicable diseases. The aim was to examine consensus and disagreement over key priorities when dealing with an outbreak of a vaccine-preventable disease (measles) in an area with strong negative sentiment towards vaccination. There is a growing body of research about vaccine hesitancy and the anti-vaccination movement [11–17]. Although vaccine hesitancy is widely researched [18–26], there is not always clear consensus about its definition. Leask et al. [27] identify five parental positions ranging from ‘unquestioning acceptors’ to ‘refusers’. Those who refuse childhood vaccinations are thought to comprise less than 2% of the Australian general population, however there are geographical clusters within some regions of Australia where the rates of vaccine objection are higher than average [28]. For the survey, we asked participants to consider a scenario in a hypothetical community known for high anti-vaccination sentiment and a high proportion of vaccine refusal.

## Methods

### Delphi process

We conducted a three-round modified Delphi process. The rationale underpinning Delphi surveys is that consensus about contentious issues carries more weight than individual opinions [29]. Anonymous data are collected from individuals, collated and then re-presented to the group to elicit further responses [30]. Delphi surveys allow a structured, iterative collection and refinement of opinions from a range of perspectives and stakeholders on complex or contentious topics [29, 30]. Participants are moved towards consensus over the course of 2 to 4 rounds through reflecting and responding constructively to the comments and positions taken by other participants. Delphi surveys have been successfully used in the Australian and international communicable disease and public health fields [31–35] and were therefore appropriate to apply in this study. We used a modified Delphi technique to allow for a qualitative analysis of panellists’ views, to explore agreements and disagreements. We used *Qualtrics*, an online survey platform, to conduct three rounds of questions. In the first round we asked participants to read the following fictional but fact-based scenario of a measles outbreak:

*There have been six notifications of measles in your area in the last week. Three are young children aged between 18 months and three years who attend a single family day-care provider, the other three are school-aged children attending two different primary schools in the same suburb. None of the children reside in the same household. Four of the children are completely unvaccinated, two partially vaccinated (both have received DTPa, but no other vaccines). This suburb is known for a geographical cluster of conscientiously unvaccinated children, and there is a fairly strong anti-vaccination sentiment in the local community with quite a number of children who are partially / selectively vaccinated, and some who are not vaccinated at all.*

We then asked participants to answer five open-ended questions (see additional file 1) designed to elicit their perspectives on what should be the priorities in addressing the hypothetical outbreak, what practical issues and challenges they would face, and what they would need from non-vaccinating members of the community to effectively manage the measles outbreak.

The process involved iterative refinement. One author (PR) reviewed the qualitative responses, identifying and grouping similar statements together. The other authors (KW and CD) reviewed a sample of the responses in order to ensure that the qualitative coding was consistent. Next, we did a descriptive analysis, first by calculating frequencies of the responses and compiling lists of the five or six most common responses for each question. The most common answers from each Round 1 question were summarised and presented anonymously to Round 2 participants, who were asked to rank in order of importance the most frequent concerns, priorities, practical issues or operational needs. Their preferences were then weighted to allow for comparison of responses. Higher ranked responses were weighted more heavily than lower ranked responses. For example, because there were five possible responses, priority 1 was given a weighting of 5, priority 2 a weighting of 4, priority 3 a weighting of 3 and so on. For a given response, the total number of times it was ranked 1 by the respondents was then multiplied by 5; the total number of times it was ranked 2 was multiplied by 4 and so on, such that a weighted total rank score was given for each response. This enabled an aggregate ranking of the responses for each question as given by the Delphi panel. Graphs for the relative (unweighted) rankings for each response can be found in the supplementary materials.

In Round 3, the final round of the survey, we provided the panel with a summary of the rankings from Round 2 and asked participants to comment on whether they agreed or disagreed with the rankings. Round 2 and 3 included a combination of Likert scales, ranking/sorting and open-ended text boxes, to allow for further explanation or comment (see additional file 1 for survey questionnaires).

### Participants

The expert panel was made up of a range of professions, all working within immunisation and communicable disease control. Email invitations were sent to 167 potential participants from all over Australia, representing a range of professions and operational levels (i.e. local, regional, state or national). These potential participants were identified through State Health Department public health unit web pages, and through networks of public health professionals to which the research team belong. As Wilkes [36] outlines, Delphi surveys typically use convenience samples, and the expertise of the panel is usually more important than the sample size.

Participants held a number of roles in immunisation and public health, including Immunisation and Child Health nurses, local area Immunisation Team Leaders and Coordinators, Public Health Physicians, district Public Health and Communicable Disease Control Directors, an executive public health professional and a public health academic (see Table 1). In each round, approximately half of respondents operated at a local or regional level of responsibility, the remainder being state-level, with one national-level participant in each round (see Table 2).

**Table 1:** Professional roles of respondents

Professional Roles	Round 1	Round 2	Round 3
Immunisation team leader / coordinator	13	10	11
Clinical nurse specialist	7	3	2
Director of Public Health Unit	6	4	7
Maternal and child health nurse	4	4	1
Immunisation nurse / registered nurse	5	1	2
Doctor / Public health physician	2	8	1
Director of Communicable Diseases Control	2	1	1
Executive public health professional	1	1	1
**Total:**	40	32	26

**Table 2:** Spheres of operational responsibility of respondents

Tier of responsibility	Round 1	Round 2	Round 3
Local	20	16	11
Regional	14	9	8
State	5	6	6
National	1	1	1
*Total respondents*	*40*	*32*	*26*

### Response rates

The response rates varied for each round (see Table 3), with 40/167 responses returned for Round 1 (response rate 24%), 32/157 returned for Round 2 (response rate 20%), and 26/136 responses returned for Round 3 (19%). The number of invitations varied from round to round because several email addresses used in Round 1 bounced (i.e. They were inactive email addresses) or because a participant had ticked the box to say they did not want to be contacted about the study in the future.

**Table 3:** Response rates of each round of the Delphi process

Delphi Round	Round 1	Round 2	Round 3
Invitations	167	157	136
Responses	40	32	26
Response rate	24%	20%	19%

## Results

### The potential for a large outbreak of measles

**Fig. 1:**
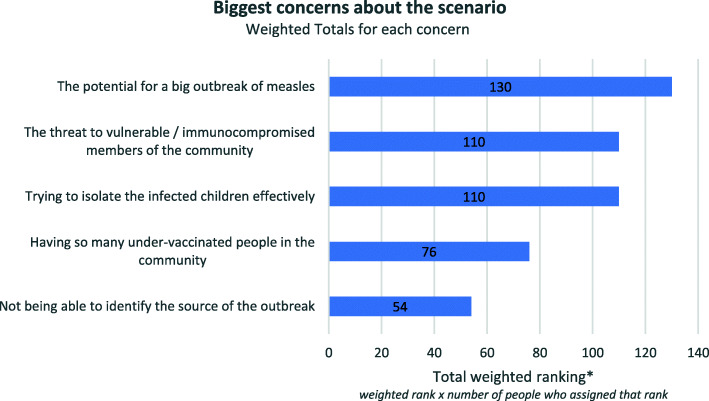
Biggest concerns about the scenario, weighted totals (Round 2)

The ranking scores for first and equal second place were very close (Fig. 1). The highest-ranked concern was the potential threat of a large outbreak of measles, followed by prioritising the isolation of infected children and protecting the most vulnerable community members, which were ranked equal second. The highest ranked option was ‘the potential for a big outbreak’, while the lowest ranked overall was ‘not being able to identify the source’, both receiving over 50% of the highest (first) and lowest (fifth) rankings respectively. Options 2–4 were more evenly spread (see supplementary graph, additional file 2).

In Round 3, the respondents indicated strongly that they agreed with these rankings, with 21/24 agreeing or strongly agreeing with the way these concerns had been ranked in the prior round. In their comments, Delphi participants noted that they agreed with the ranking of priorities because the focus is on reducing the risk of transmission and protecting vulnerable members of the community:

Sensible ordering of concerns – emphasis on protecting people, especially vulnerable people.

(Respondent with regional sphere of responsibility)

It follows principles of public health response - which focuses on the risk of transmission and preventing this and focuses on the highly vulnerable group who are unable to protect themselves but are more likely to have severe disease outcomes.

(Respondent with local sphere of responsibility)

Commenting on the importance of stopping the spread of the disease, several participants mentioned that parents are usually fairly compliant when it comes to isolating infected children:

The rankings generally reflect my concerns and their priority. I agree (rather than strongly agree) with rankings because my experience is that generally the families of known affected children are generally compliant with advice about isolation.

(Respondent with regional sphere of responsibility)

In my experience parents comply with isolating children once advised. Vaccine refusers are particularly compliant perhaps because they understand the implications to their position if they don't and are responsible for further transmission.

(Respondent with state sphere of responsibility)

The small number of participants who indicated they disagreed with the ranking did so because they believed that the priorities and concerns overlap, for example:

Many of these issues are simply different facets of the same fundamental challenge. They are all strongly linked. But a big outbreak is the consequence of concern and the other issues are either drivers or barriers or smaller scale issues. So I agree but all responses are really valid concerns!

(Respondent with state and national spheres of responsibility)

There did not appear to be a patterned difference in responses to the question about key concerns between participants who held positions of national, regional or local levels of responsibility. The small number of respondents who disagreed or chose neither agree nor disagree, commented that the threat to vulnerable community members and containing the spread of the disease (by isolating those affected) should be higher priority. For example:

This degree of clustering of under vaccinated children is unusual and requires long term action, but not as urgently as concerns about spread and need for isolation of cases.

(Respondent with regional sphere of responsibility)

Just because there may be parents who do not wish to vaccinate their children doesn't mean they would not engage in isolating their infected child. I think point 3 and 4 should be higher in rank.

(Respondent with state sphere of responsibility)

I believe ‘The threat to the vulnerable/immunocompromised’ should be of most concern as this is the group who most ‘benefits’ from Herd Immunity.

(Respondent with local sphere of responsibility)

### Isolating infected children

**Fig. 2:**
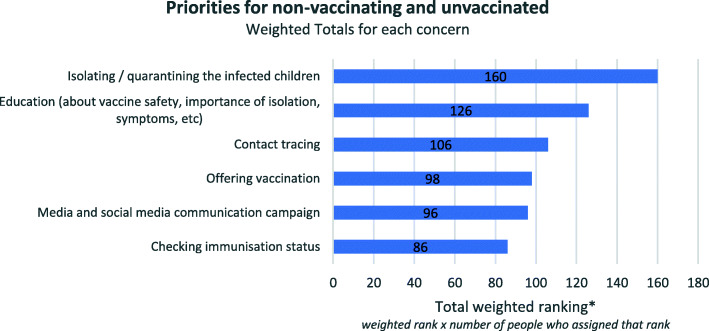
Priorities for non-vaccinating and unvaccinated, weighted totals (Round 2)

Participants in Round 2 were asked to rank the most common responses to the question: *What would be your priority for the non-vaccinating or unvaccinated members of your community in this scenario?* Figure 2 shows that isolating or quarantining infected children was the highest ranked priority, however support for the other options was more evenly spread.

The highest ranked priority for the non-vaccinating and unvaccinated, ranked number one by over 60% of participants, was isolating infected children. Options 2–6 were ranked more evenly (see supplementary graph, additional file 3). When reflecting on the high priority of isolating infected children, Delphi panel members emphasised that the most important thing to do was to *minimise the potential for the spread of the disease*. For instance:

Need to isolate and contact tracing to reduce further spread.

(Respondent with local sphere of responsibility)

The biggest priority is minimising the spread of measles in a largely unvaccinated population. Isolating and quarantining will be more effective in the short term. Vaccination is important but takes longer to be effective.

(Respondent with local sphere of responsibility)

Education was ranked second, with participants outlining that it was important for this community to be educated about the risks of not vaccinating and to keep them informed about what signs and symptoms to look out for:

Also need to do a lot of education about recognising the symptoms of measles, self-isolation and presentation to healthcare early in course of illness for testing.

(Respondent with state sphere of responsibility)

Interestingly, checking immunisation status was ranked the lowest. When asked to comment on why they thought: *“Media and social media communication campaign”* and *“Checking immunisation status”* were both ranked lower priorities than the other options, panellists suggested that other priorities in the list are a more urgent during an outbreak. They also noted it was likely that all options would happen simultaneously and that in an outbreak situation the priority target populations are those infected or in contact with the disease. For example:

These are both equally important measures but obviously not 1st response.

(Respondent with local sphere of responsibility)

These can both happen down the track – It is not a priority.

(Respondent with local sphere of responsibility)

Both media communication and checking immunisation status are broader, ‘slower’ and less targeted interventions. The key populations to target to mitigate or prevent a large outbreak are the cases and contacts.

(Respondent with state and national spheres of responsibility)

It depends on what the media campaigns are targeting, if it is for public awareness regarding an outbreak then the priority should be higher but if it is trying to improve vaccination rates in a population that already has very strong preformulated opinions on vaccination it is not going to make much of a difference and therefore a lower priority.

(Respondent with local sphere of responsibility)

Even though our results indicate that participants ranked a wider range of concerns as being key priorities, the tier of operational responsibility does not appear to have influenced their responses to this question.

### Encountering (and countering) mistrust

In Round 1 we asked participants to outline the practical issues they would face in an outbreak, relating to the non-vaccinating community. Common responses included: mistrust, the need for communication, difficulties in isolating/excluding, the strong negative sentiment regarding vaccination. The following quotes illustrate the dominant themes arising from the first round of the survey:

Misinformation about VPDs [vaccine preventable diseases] and vaccines and mistrust in health professionals.

(Respondent with local and regional spheres of responsibility)

The need for a rapid response and the tension between this and the need for an iterative, engaged, nuanced communication with individual families and others.

(Respondent with State sphere of responsibility)

Enforcing isolation during incubation period - especially if they are a large number in the community and their concern of loss of income having to take sick leave, etc.

(Respondent with local sphere of responsibility)

**Fig. 3:**
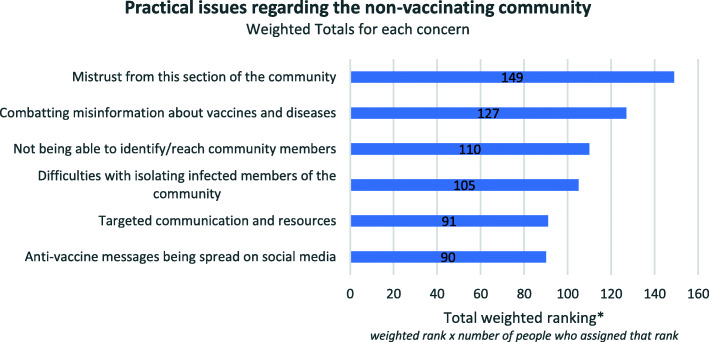
Practical issues faced regarding the non-vaccinating community during an outbreak, weighted totals (Round 2)

The highest ranked practical issue faced by Round 2 participants was encountering mistrust from the non-vaccinating members of the community. Similarly to the previous question, the rankings for the other options were relatively evenly spread. Figure 3 indicates the order of importance, as ranked by participants. The relative ranking of responses for this question was more evenly spread than other questions, with all ranked responses being below 35% (see supplementary graph, additional file 4) suggesting that all options are important to the extent that they are different facets of the problem of gaining the trust of and communicating constructively with the non-vaccinating community.

Several respondents talked about the need to combat misinformation about vaccines, but the general consensus was that this kind of public campaign was not high priority during an outbreak. Social media was regularly mentioned in regard to spreading public health information about the outbreak, to educate the public about looking out for symptoms and isolating:

Social media to notify the community of real and inherent danger to the population

(Respondent with regional sphere of responsibility)

Communication and using social media would be next step to enable other vulnerable community groups and those only partially immunised to be offered immunisation or immunoglobulin to prevent them getting infected.

(Respondent with regional sphere of responsibility)

Respondents to Round 3 showed strong support for the rankings provided by the Round 2 panellists, with the majority (18/24) agreeing or strongly agreeing with the rankings outlined in Fig. 3. Two out of 24 neither agreed nor disagreed and 4 out of 24 disagreed. Once again, the participant’s tier of responsibility did not influence their response. In their comments those who did not agree reasoned that it was not productive to pressure people into immunising their children – the focus should be on making them aware of the opportunity to do so. For example:

If we assume non vaccinating community means vaccine refusers rather than vaccine hesitant people, then there is little utility in gaining trust or combatting misinformation to this community. I would be more concerned about anti vaccination messages being spread to non-vaccinated community who are not vaccine refusers.

(Respondent with state sphere of responsibility)

Non vaccinators are well informed and have made a choice. Options for people to change their mind and vaccinate should be easy.

(Respondent with local sphere of responsibility)

A small number of respondents mentioned that they preferred a different ranking of priorities, pointing out that combatting misinformation would be a lower priority than practical things like contacting potentially infected community members. For example:

Mistrust may be present but ought not be presumed to be a limiting difficulty. Providing sound practical information is more important in this setting that trying to dismantle contrary beliefs, and an adversarial approach in this context may even fuel unhelpful ideas.

(Respondent with state sphere of responsibility)

I would rate combatting misinformation etc. lower e.g. below current #5 [“targeted communication and resources”]. Need to reach community members, isolate infected and quarantine exposed before tackling long-held beliefs and misinformation.

(Respondent with regional sphere of responsibility)

In Round 3 we asked respondents to comment on what strategies in their experience have proved the most successful for countering mistrust among non-vaccinating parents during an outbreak. Recurring responses to this question were the need for patience and calm education. Panellists suggested it was important to highlight to parents the serious complications that can arise from the disease. Further, they suggested that it is important not to get into arguments with non-vaccinating members of the community, but instead to be offer reassurance, provide accurate information and to acknowledge different beliefs. For instance:

Not arguing with non-vaccinators and always be truthful in your answers

(Respondent with local sphere of responsibility)

Reassurance, patience, education may help with those people that are still ‘sitting on the fence’ about vaccination.

(Respondent with local sphere of responsibility)

Recognising and acknowledging people’s beliefs, however conveying facts and not getting drawn into a discussion or arguments. Consistent and clear media messaging.

(Respondent with regional sphere of responsibility)

### Practitioners want a willingness to listen and cooperate

**Fig. 4:**
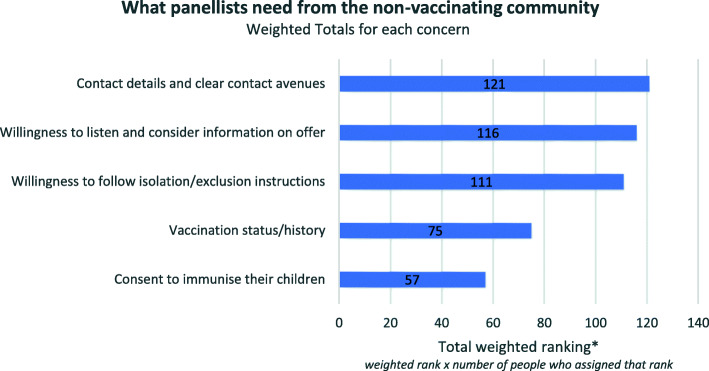
What panellists need from the non-vaccinating community, weighted totals (Round 2)

In an outbreak scenario ‘contact details and clear contact avenues’ were ranked the most important, in terms of what the panellists would need from the non-vaccinating community. However, it was not ranked significantly higher than 2 and 3: a willingness to listen and consider information, and willingness to follow isolation instructions. The rankings for this question were relatively evenly spread. Notably, ‘consent to immunise their children’ was ranked the lowest (Fig. 4).

The lowest ranked option, with over 50% placing this last, was ‘consent to immunise their children’ (see Additional file 5). Related to the highest concern being the potential for a large outbreak, the need for clear contact tracing avenues and a willingness among non-vaccinating parents to follow isolation instructions and consider information on offer are both vital for public health workers in managing the spread of the disease. Several respondents mentioned that in their experience, cooperation from parents is variable. For example:

Varied responses across the acceptance/refusal spectrum. Some are willing to listen, discuss and maybe vaccinate or partially vaccinate if given the time to discuss and express their concerns. Others would not get into the discussion and indicate that their decisions are final. Cooperation ranges from being willing to engage in a discussion, to partially vaccinate or get a referral to a specialised service.

(Respondent with local sphere of responsibility)

It has varied at different times and in different communities – sometimes they are quite cooperative, other times they have been very evasive/impossible to engage. That may be the reason there is such a variation in responses, if respondents have had only one or two encounters, they may have been very positive or very negative.

(Respondent with state sphere of responsibility)

Importantly, the panel identified the importance of being able to identify and differentiate between “vaccine refusers” and “vaccine hesitant”. They characterised vaccine-hesitant community members as being more likely than vaccine-refusers to listen and cooperate with health authorities. Several pointed out that trying to engage with those who have ‘made up their mind’ is unlikely to help, as the following responses illustrate:

If parents are vaccine refusing, they do not wish to engage in any way. They are not interested in information that does not support their stance. Vaccine hesitant parents are generally cautious and sometimes confronting, but they will at least ask questions and request information.

(Respondent with state sphere of responsibility)

On the rare occasions we engage with non-vaccinating parents we explain the rationale for our views and generally end up agreeing to disagree about vaccination, but they accept requirements for isolation, exclusion.

(Respondent with regional sphere of responsibility)

Limited communication and often confrontational from non-vaccinating community, for those who are just unsure, they are much more open and ask more questions and want discussion.

(Respondent with regional sphere of responsibility)

The need to treat people with respect, be empathetic and listen was also raised by a number of respondents. For example:

The core of non-vaccinators who are intransigent is really quite small - around 1%. Those who are hesitant but non-vaccinating is larger and they are influenced by the core group but open to other trusted individuals, including GPs, nurses and others. The first group are very hard to deal with and should largely be left alone. The latter group takes work but can be cooperative with the right messages and approaches; empathetic, flexible, engaged and listening.

(Respondent with state sphere of responsibility)

### Communicating with the community

Panellists made a distinction between vaccine refusers and parents with lots of concerns and questions and suggested that in an outbreak, focusing on the former is not urgent. Respectful conversations about immunisation were seen as important. Respondents acknowledged that there are structural barriers to accessing vaccination, and that different strategies are required for different groups. For instance:

These are the general priorities from my perspective. Of course misinformation via social media is an issue, but it’s a long-term, complex issue and unlikely to be an immediate priority, especially given the fact that it is promulgated by, and engaged with, the most ardent anti-vaxxers. And in this scenario, we really want to reach the ‘hesitant’ and those with logistic, awareness, financial or other challenges to vaccination.

(Respondent with state and national spheres of responsibility)

While some are totally mistrusting of vaccines, not all under-immunised or non-immunised people are due to anti-vaccination. Some just require time the opportunity to ask questions and time to digest the answers. This is often not given in acute or primary health care.

(Respondent with state sphere of responsibility)

Some participants had suggestions for how to best engage with the community during an outbreak, including the need for engaging and positive messages. For example:

Messaging must be tailored, realistic, iterative and emotionally engaging. Finding a trusted ‘broker’ from within the community can be very powerful. A generic, bland, classic ‘government’ FAQ is probably the least effective but may have widest reach. I’m increasingly of the opinion that government engagement via blogs, Twitter, Facebook, open house and other more innovative approaches is probably more robust than website/pamphlet approaches.

(Respondent with state sphere of responsibility)

Focus on delivering positive messages, simple and accurate information and identifying their immediate concerns. Explaining why outbreak has occurred. Potential health issues for their children.

(Respondent with local sphere of responsibility)

## Discussion

The public health professionals who took part in this study identified several key priorities when controlling a measles outbreak in a community with low levels of vaccine acceptance. Their primary concerns for containment and contact tracing are consistent with the need to initiate prompt public health actions and avoid a large-scale outbreak [1]. Vaccinating unvaccinated children was uniformly ranked by Australian public health professionals, across a variety of roles and levels of responsibility, as a comparatively *low* priority. Respondents acknowledged that it was not productive to pressure people into immunising their children, and what they really needed from non-vaccinating community members was willingness to cooperate with contact tracing activities and to adhere to isolation instructions.

These concerns and requirements are somewhat at odds with the media and political responses historically seen in such situations. Media coverage and public discourse surrounding recent vaccine preventable disease outbreaks such as measles and pertussis have tended to focus on vaccine refusal and the need for vaccination, rather than the immediate need for cooperation with contact tracing and isolation [37–40]. The headlines generated often heighten the politicisation of the issue, leading to policies that, while appearing popular with the media, may be at odds with operational needs and the evidence. Negative public discourse, inflamed by the mainstream media, could make the task of outbreak control more difficult. Recently in Australia, for example, the Prime Minister announced a contract with a global pharmaceutical company that would see a COVID-19 vaccine produced and made available in Australia. Citing his responsibility for introducing Australia’s mandatory childhood vaccination policies in 2017, the Prime Minister told the media that he expected the vaccine “to be as mandatory as you could possibly make it” and that medical grounds “should be the only basis” for exemption [41]. Contrary to this, there is a growing body of evidence that mandatory vaccination does not encourage vaccine refusers to change their mind, and in many cases only serves to strengthen their resolve. Furthermore, mandates tend to push those who are vaccine-hesitant (as opposed to rejectors) toward outright rejection [42, 43]. While the Prime Minister later backtracked on these comments, others have taken up the call for mandatory COVID-19 vaccination in Australia [44].

In a measles outbreak scenario like the one we described for our study, the short-term goals are about health protection in order to minimise the spread of the disease. Media campaigns can be useful in an outbreak situation if they are to focus on the things like increasing public awareness about the disease and the importance of isolation, contact tracing and the risk posed to vulnerable members of the community. Some panellists encouraged the use of social media for its ability to reach a large audience quickly, informing them of the outbreak and practical measures they need to take. However, several panellists were critical of the way misinformation about vaccines can spread rapidly on social media. Research has shown that organisations that promote vaccination face significant challenges such as quickly evolving situations and limited resources for addressing the volume of misinformation on social media, leaving some reluctant to engage significantly using this medium [45]. Steffens et al. suggest that vaccine-promoting organisations can and should use social media to directly address vaccine misinformation, crafting their responses to target the “silent” audiences who watch social media but don’t necessarily post. They suggest refutations should be straightforward and succinct and where possible pair scientific evidence with stories that resonate with the intended audience [45].

While our participants ranked addressing the spread of anti-vaccine messages on social media as a low priority during an outbreak, some participants in our study suggested that more individualised approaches were preferred to blanket media and social media responses, and that social media does have a role to play in disseminating information quickly. Targeting the hesitant parents (rather than those who are vaccine-refusing) to discuss their concerns on a more individual level may be more successful in such a scenario [46]. Parents with questions could be encouraged to talk with their healthcare provider. Evidence-based information tools and communication strategies have been developed to aid vaccination conversations between parents and providers [47, 48]. Training is available for Australian healthcare providers under this approach in how to identify the level of vaccine hesitancy and tailor their discussion approach accordingly [49, 50]. While this intervention addresses issues with vaccine hesitancy, broader issues around willingness to comply with isolation and exclusion requirements are not addressed. The advent of COVID-19 has brought this issue to the fore. Development of similar information and communication strategies about self-isolation and exclusion are becoming increasingly necessary.

Managing any communicable disease outbreak involves public health professionals and members of the public working collaboratively. For a vaccine-preventable disease outbreak in a community with high levels of resistance to vaccines, there are potential barriers to effective collaboration based on personal belief and mistrust. The participants described ways to address these barriers in terms of both *what* actions could/should be taken and *how* those actions should be implemented. There was some disagreement among the panel as to operational priorities. Most ranked combatting mistrust and misinformation highly, with a small number disagreeing. Rather than explicitly focusing on mistrust, a handful of participants maintained a preference for focusing on establishing good lines of communication, providing practical information and facilitating the isolation of probable cases. Recent research in Australia shows that mistrust and the use of alternative sources of information are a known phenomenon among non-vaccinating parents, who often go to great lengths in their self-directed investigation into vaccination and therefore feel very confident in their views [42, 43, 51, 52]. This research also found that a very small number of parents mentioned that they *may* be willing to re-consider in an outbreak scenario [43]. Therefore, a broad emphasis on “combatting” misinformation targeted at vaccine-refusers may not be an effective approach in such a scenario, especially given that it has been shown that people with non-vaccination views often become more steadfast in those views when presented with vaccine supportive information [53].

Finally, the participants had a high level of awareness of the nuance surrounding vaccine hesitancy and refusal. In responding to the scenario they operationalised this knowledge in how they described their approach to engaging and interacting with these different groups – often emphasising the need for respect, calm, patience and empathy, in line with the best evidence on how to communicate with vaccine hesitant and refusing parents [27, 47, 48, 54]. Participants spoke of countering mistrust by providing reliable information and not adopting an adversarial approach when communicating with non-vaccinating members of the community, a crucial factor when working with parents who may have had previous adversarial interactions with the health system [43, 51]. Their reported experiences of responses from non-vaccinating members of the community to this approach varied; some participants felt that vaccine-hesitant parents were more likely than vaccine-refusers to listen and have a conversation with health authorities, however many suggested both groups were willing to cooperate in their experience.

## Conclusions

Our findings provide a deeper understanding of the challenges faced during an outbreak and priorities for communicating with communities with low vaccination coverage. For health professionals in our survey, highest ranked concerns and priorities were not about increasing vaccination rates or tougher measures like vaccination mandates. Instead, their focus was, firstly, on ensuring the disease does not spread far; and secondly, keeping lines of communication open with vaccine-hesitant and vaccine-refusing parents. The respondents to our survey offered nuanced understandings of the complexity of vaccine decisions – and their experiences of communicating with non-vaccinators showed an appreciation of the need to listen, be empathetic, and not get involved in arguments or trying to change people’s minds. Rather, focusing on contact tracing and adhering to isolation guidelines is a better approach, with some respondents reporting that non-vaccinating parents can be quite willing to isolate and follow public health instructions, and others reporting their experience as variable.

A hypothetical and idealised outbreak scenario was used to drive this Delphi study. Limitations of our study are that we did not explore what the concerns might be in an outbreak scenario amongst a community with higher levels of vaccine coverage. During a real outbreak, differences in social and epidemiological context factors may require a different response from decision makers. Future research might make a comparison between how public health officials respond to the threat of a measles outbreak in an area where there is relatively high vaccine coverage.

The relatively low response rate is also a limitation, although the decreasing level of responses with each round is common with Delphi surveys, especially among busy professionals. Also, we must be clear that while the study results have relevance for the management of COVID-19, our study was conducted before COVID-19 was known to exist.

Strategies to help frontline workers communicate effectively with all members of the community would assist in outbreak control. This would include guidance on the effective use of social media to rapidly disseminate information and address misinformation. More broadly, guidance could be developed on how to engage with the media on the framing of an outbreak, such that they focus on the things most useful to the public health management of the outbreak – contact tracing and isolation – rather than focusing on polarising issues like vaccine refusal, which are largely unhelpful to public health professionals and community members in these situations. Similarly, early engagement with political actors on the evidence base (or lack thereof) supporting different policy measures such as vaccination mandates might help avoid politicisation of the issues associated with such an outbreak before they become enacted. Our findings have implications beyond measles outbreaks. In the new public health context where COVID-19 vaccines are being rolled out across the globe – with high levels of public uncertainty, media coverage and politicisation – the empathetic approach and clear messaging around prioritising of contact tracing and isolation described by the participants in this study are highly relevant. It will be important to achieve a high uptake of the vaccines in order to attain herd immunity. Our findings could be used to inform guidance and communication strategies for the other key actors, namely the media and the public.

## Supplementary Information

**Additional file 1.** Questionnaires for Rounds 1–3 of the Delphi survey. Questionnaires for Rounds 1, 2 and 3 of the survey.

**Additional file 2.** Biggest concerns about the scenario, Relative rankings (Round 2). Graph showing relative ranking of responses.

**Additional file 3.** Priorities for non-vaccinating and unvaccinated, relative rankings (Round 2). Graph showing relative ranking of responses.

**Additional file 4.** Practical issues faced, Relative rankings (Round 2). Graph showing relative ranking of responses.

**Additional file 5.** What panellists need from the non-vaccinating community, Relative rankings (Round 2). Graph showing relative ranking of responses.

## Data Availability

The dataset generated and analysed during this study is not publicly available due to the conditions of ethics approval and to protect the privacy of individual participants.

## Abbreviations

AIR: Australian immunisation register
COVID-19: The strain of coronavirus that causes coronavirus disease 2019
DTPa: Diptheria, tetanus, pertussis-attenuated vaccine
FAQ: Frequently asked questions
GPs: General practitioners
PHU: Public health unit
SARS-CoV-2: Severe acute respiratory syndrome coronavirus 2
VPDs: Vaccine preventable diseases
WHO: World health Organisation
