# Supplementary material for: Public health practitioner perspectives on dealing with measles outbreaks if high anti-vaccination sentiment is present
Source: BMC Public Health. 2021 Apr 9;21:578. doi: 10.1186/s12889-021-10604-3 (PMC8032458; doi:10.1186/s12889-021-10604-3)
Supplement: Supplementary file 1 — Additional file 1. Questionnaires for Rounds 1–3 of the Delphi survey. Questionnaires for Rounds 1, 2 and 3 of the survey. [file 12889_2021_10604_MOESM1_ESM.pdf]

Additional File 1: Questionnaires for Rounds 1-3 of the Delphi survey

ROUND 1: Questions posed to participants

1. What concerns would keep you awake at night in this scenario?
2. What would be your priority for the non-vaccinating community in this scenario?
3. What are the practical issues that you face regarding the non-vaccinating community in this area in this scenario?
4. What would you need from the non-vaccinating community in this scenario? (e.g. information, permissions, clear contact avenues etc.)
5. What are the challenges you face in getting what you need from the non-vaccinating community in this scenario?

ROUND 2: Summary of R1 and questions posed to participants

A Delphi survey is a method used to create commentaries on a specific topic over multiple rounds with the aim of building consensus across experts. In the first survey, you were given a scenario and asked to respond to five questions about it. In Round 2 you will be asked to rank the most common responses we received to those questions in order of importance.

Summary of Round One

Participants in Round 1 were invited to share what worried them most about the measles outbreak scenario. Common responses included: the risk of a serious outbreak of measles, the threat to vulnerable members of the community, and not being able to effectively exclude individuals who had contracted measles. We also asked you to outline what the top priorities were for the non-vaccinating members of the community. Respondents mentioned the importance of checking immunisation status and offering vaccination, contact tracing, and isolating infected individuals. Other themes included the importance of education and the need for communication campaigns. The following quotes from participants indicate some of the key priorities:

> *"To convince families of the urgent need for vaccination and then to put in place arrangements for vaccination."*
> *"Also need to do a lot of education about recognising the symptoms of measles, self-isolation and presentation to healthcare early in course of illness for testing."*
> *"Increase communications via social media in relation to presentation of disease"*

In Round 1 we asked participants to outline the practical issues regarding the non-vaccinating community. Common responses included: mistrust, the need for communication, difficulties in isolating/excluding, the strong negative sentiment regarding vaccination. The following quotes indicate the dominant themes arising from the first survey:

> *"Misinformation about VPDs and vaccines and mistrust in health professionals."*
> *"The need for an iterative, engaged, nuanced communication with individual families and others."*
> *"Enforcing isolation during incubation period - especially if they are a large number in the community and their concern of loss of income having to sick leave, etc."*

Participants told us what they would need from non-vaccinating parents in this scenario. Responses included: vaccination status and history, consent to vaccinate, a willingness to listen and cooperation with public health recommendations. Lastly, we asked you to tell us about the challenges you face in getting what you need from the non-vaccinating community in this scenario. Answers to this question included being faced with defensiveness and being distrusted as health professionals, for example:

> *"Skills to get the information across to them as some can be very defensive and not listen."*
> *"This group generally has a low regard and intense distrust of doctors, drug companies and science"*
> *"Vaccine refusers sometimes believe it is better to get the disease naturally to improve their immunity to particular diseases"*

Questions

1. What is your professional title? (Please enter your current role below)
2. What is your sphere of responsibility? (Local, regional, state, national)

3. Please read the scenario and then answer the questions below. The options provided for each question were answers given by participants in Round 1.

3.1. Having reread the scenario and seen the responses of other panel members, what worries you the most about this scenario? Please drag and drop the following options to rank them in order of importance, with the most important at the top.

- Trying to isolate the infected children effectively
- Not being able to identify the source of the outbreak
- The potential for a big outbreak of measles
- The threat to vulnerable / immunocompromised members of the community
- Having so many under-vaccinated people in the community

Are there any other significant concerns that should be on this list?

3.2. In the first survey we asked "What would be your priority for the non-vaccinating or unvaccinated members of your community in this scenario?". Below are the six most common responses. Please drag and drop the following options to rank them in order of importance, with the most important at the top.

- Media and social media communication campaign
- Offering vaccination
- Education (about vaccine safety, importance of isolation, symptoms, etc)
- Isolating / quarantining the infected children
- Checking immunisation status
- Contact tracing

3.2a. Please explain your ranking decisions in the text box below.

3.3. What are the practical issues that you would face in your position regarding the non-vaccinating community in this scenario? Please drag and drop the following options to rank them in order of importance, with the most important at the top.

- Mistrust from this section of the community
- Targeted communication and resources
- Difficulties with isolating/excluding infected members of the community
- Combatting misinformation about vaccines and diseases
- Anti-vaccine messages being spread on social media
- Not being able to identify/reach community members

3.3a. Please explain why you ranked them in this order.

3.4. What would you need from non-vaccinating parents/carers in this scenario? Please drag and drop the following options to rank them in order of importance, with the most important at the top.

- Contact details and clear contact avenues
- Vaccination status/history
- Willingness to follow isolation/exclusion instructions
- Consent to immunise their children
- Willingness to listen and consider information on offer

3.5. In Round 1 we asked: "What are the challenges you face in getting what you need from the non-vaccinating community in this scenario?" Please indicate below the extent to which you agree with the most common responses.

	Strongly agree	Agree	Neither agree nor disagree	Disagree	Strongly disagree
a) Misinformation/Misunderstanding about the importance of vaccination	O	O	O	O	O
b) Unvaccinated and under-vaccinated are difficult to identify	O	O	O	O	O
c) Vaccine-refusing parents can be uncooperative / mistrusting / unwilling to engage with public health	O	O	O	O	O
d) Challenges with informing and educating	O	O	O	O	O
e) Hostility and defensiveness	O	O	O	O	O
f) Some want to expose their child to measles for 'natural immunity'	O	O	O	O	O

4. Are there any other significant challenges that have not been addressed? Please add further comments in the box below.

Round 3: Summary of R2 and questions posed to participants

Participants in Round 2 ranked the most common responses from the panel in order of most important to least important. The preferences were weighted to assist in understanding how respondents prioritised the options provided. The total for each given response was counted, each rank was totalled, and the results weighted for priority. For example, where there were five possible responses, priority 1 was given a weighting of 5, priority 2 a weighting of 4, priority 3 a weighting of 3 and so on. For a given response, the total number of times it was ranked 1 was then multiplied by 5; the total number of times it was ranked 2 was multiplied by 4 and so on, such that a weighted total rank score was given for each response. This enabled an aggregate ranking of the responses for each question as given by the Delphi panel.

Having had time for reflection and having read the scenario again, please consider the rankings provided by the Delphi panel and answer the following questions. We expect this survey to take approximately 5-10 minutes. Thank you in advance for your time.

1. What is your professional title? (Please enter your current role below)
2. What is your sphere of responsibility? (Local, regional, state, national)
3. The first question asked what worried participants the most about the scenario.

The Weighted rankings were as follows:
1) The potential or a big outbreak of measles
2) Trying to isolate the infected children effectively (Ranked equally as second)
2) The threat to vulnerable / immuno-comprised members of the community
3) Having so many under-vaccinated people in the community
4) Not being able to identify the source of the outbreak

3.1. Indicate the extent to which you agree with the ranking of these responses.
strongly agree / agree / neither agree nor disagree / disagree / strongly agree

3.1A. Please indicate in the text box below why you feel this way, or if anything significant has been overlooked.

Priorities for non-vaccinating and unvaccinated
In the previous round, our participants were asked to rank the most common responses to the question *What would be your priority for the non-vaccinating or unvaccinated members of your community in this scenario?* As you can see from these graphs, isolating/quarantining infected children was the highest ranked priority, however support for the other options were more evenly spread.
In order of most important to least the Delphi panel in Round 2 ranked the options as follows:

1) *Isolating / quarantining the infected children*
2) *Education (about vaccine safety, importance of isolation, symptoms, etc)*
3) *Contact tracing*
4) *Offering vaccination*
5) *Media and social media communication campaign*
6) *Checking immunisation status*

We asked participants to explain their ranking in a text box. 27 out of 32 participants responded to this question, with the predominant response being that the most important thing to do was to ***minimise the potential for the spread of the disease***, especially through isolating and quarantining. For instance:

> *"Need to isolate and contact tracing to reduce further spread"*
> *"The biggest priority is minimising the spread of measles in a largely unvaccinated population. Isolating and quarantining will be more effective in the short term. Vaccination is important but takes longer to be effective."*

A few people made the point that checking immunisation status was low priority or something that could be done in parallel with other measures. Interestingly, the second-highest ranked priority, differed depending on the job title. For nurses and public health directors, "Contact tracing" was ranked second most-highly, while for doctors and immunisation coordinators, "Education" was considered more important.

3.2. "Media and social media communication campaign" and "Checking immunisation status" were both ranked lower priorities than the other options. Can you comment on why you think this might be the case?

We asked participants in Round 2 to rank the practical issues they face regarding the non-vaccinating community in relation to this imagined outbreak scenario. In order of importance you ranked the practical issues from high to low (when weighted)

1) *Mistrust from this section of the community*
2) *Combatting misinformation about vaccines and diseases*
3) *Not being able to identify/reach community members*
4) *Difficulties with isolating/excluding infected members of the community*
5) *Targeted communication and resources*
6) *Anti-vaccine messages being spread on social media*

The following quotes illustrate how participants justified their ranking decisions:

"Already a mistrusting community so combating their beliefs about the disease and vaccine will be difficult."
"In my experience when this has occurred in the past, there was a denial about the dangers of measles to the community and under reporting of further cases in a non - vaccinating community."
"An outbreak may not necessarily be the time to expend limited resources in a combative approach to strongly held beliefs, and indeed may risk driving people away from public health (& other) workers who are managing the outbreak. I've therefore ranked trying to reach the community and gain their trust as the highest priority items in managing the outbreak. This contact and trust is need to effect isolation/exclusion."
"I don't think changing minds or debating with anti-vax elements is very productive during an outbreak. i would focus on those needing vaccination because of simple inattention: during an outbreak there will be enough information to convince them"
"Targeting individuals with the right resources and messages will be harder because of the anti vaccine messages on social media"
"Often this group spread inaccurate information in the belief that they are providing sound information, this spreads quickly through social media. Many people believe much of what they read online with no concern for the accuracy of the information."

> *"Our communications should address the facts to start with and how to access vaccinations, but longer term communications would address the misinformation and anti-vaccine messages being spread on social media."*

3.3A. Indicate the extent to which you agree with the ranking of these responses.
strongly agree / agree / neither agree nor disagree / disagree / strongly agree
Please indicate in the text box below why you feel this way, or if anything significant has been overlooked.

3.3B. Mistrust from the non-vaccinating community was ranked the highest. In your experience, what strategies (if any) have proved most successful as a means of countering mistrust among non-vaccinating parents during an outbreak?

In Round 2 we asked the panel to rank the extent to which they agreed with the most common responses to Round 1 answers to the question *'What are the challenges you face in getting what you need from the non-vaccinating community in this scenario?'* Overall, there was strong support for all the statements, with 4 of the statements generating either strongly agreed or agreed from 25 or more respondents. The two statements that participants disagreed with the most were: "unvaccinated and under-vaccinated are difficult to identify" and "some want to expose their child to measles for 'natural immunity', however it is important to note that over half of respondents also marked these as Agreed or Strongly agreed.

29 of 32 respondents in Round 2 strongly agreed or agreed with "vaccine-refusing parents can be uncooperative/mistrusting/unwilling
28 of 32 respondents strongly agreed or agreed with "misinformation/misunderstanding about the importance of vaccination".
27 of 32 respondents strongly agreed or agreed with "challenges with informing and educating"
25 of 32 respondents strongly agreed or agreed with "hostility and defensiveness"
20 of 32 respondents strongly agreed or agreed with "some want to expose their child to measles for 'natural immunity' "
17 of 32 respondents strongly agreed or agreed with "unvaccinated and under-vaccinated are difficult to identify"

3.3C. In your experience, how much cooperation do you receive from the non-vaccinating community?

We asked our panel to rank in order of importance the sorts of <u>things they would need from the non-vaccinating community in this scenario.</u> From highest to lowest, you ranked them as follows:

1) *Contact details and clear contact avenues*
2) *Willingness to listen and consider information on offer*
3) *Willingness to follow isolation/exclusion instructions*
4) *Vaccination status/history*
5) *Consent to immunise their children*

3.4. Does the list above capture all the different types of support that the non-vaccinating community can provide to health authorities during an outbreak?
YES / NO / UNSURE / OTHER?
If you selected Unsure or Other above, please explain further in the text box below.
