# Supplementary figures and images for: Public health practitioner perspectives on dealing with measles outbreaks if high anti-vaccination sentiment is present
Source: BMC Public Health. 2021 Apr 9;21:578. doi: 10.1186/s12889-021-10604-3 (PMC8032458; doi:10.1186/s12889-021-10604-3)

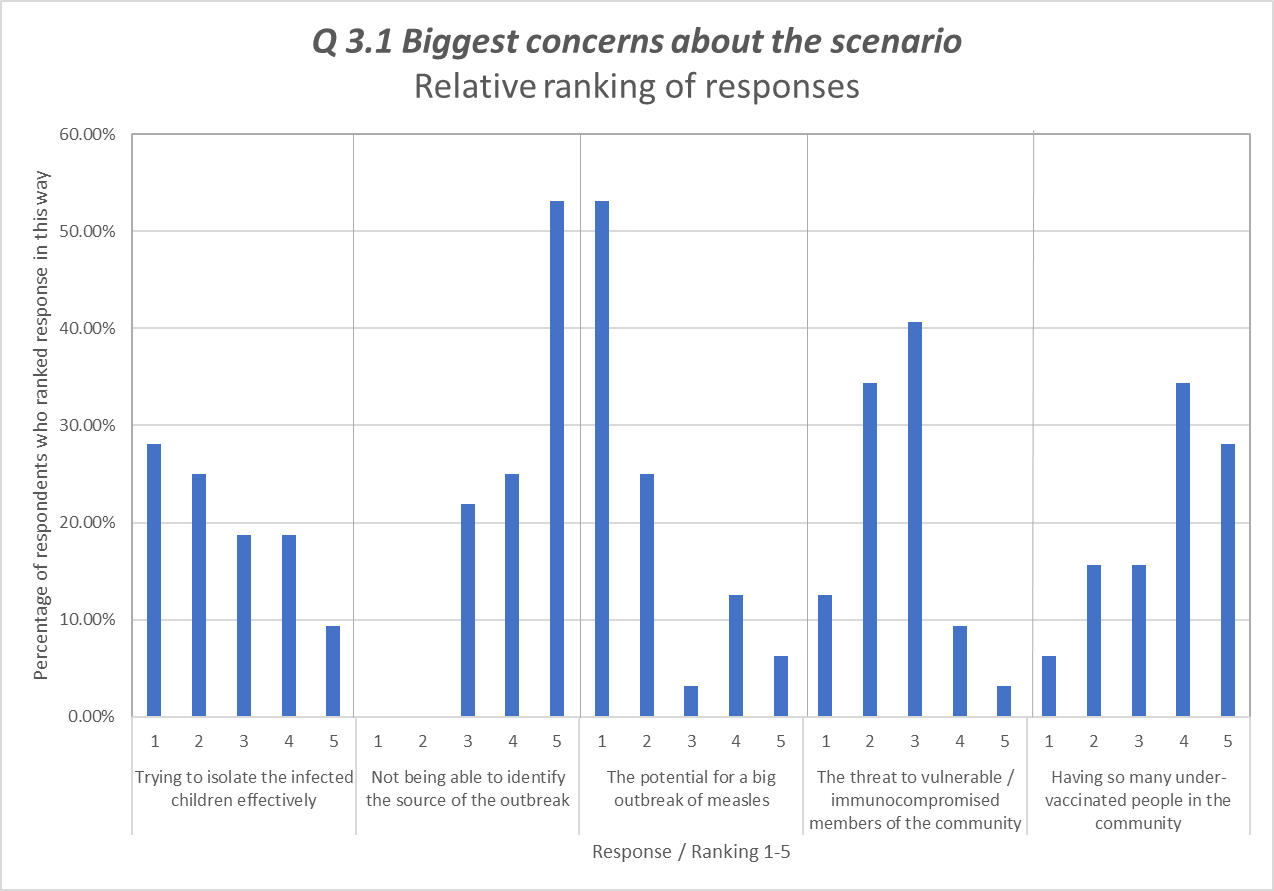


Additional File 2: Biggest concerns about the scenario, Relative rankings (Round 2)

Supplement: Supplementary file 2 — Additional file 2. Biggest concerns about the scenario, Relative rankings (Round 2). Graph showing relative ranking of responses. [file 12889_2021_10604_MOESM2_ESM.docx]

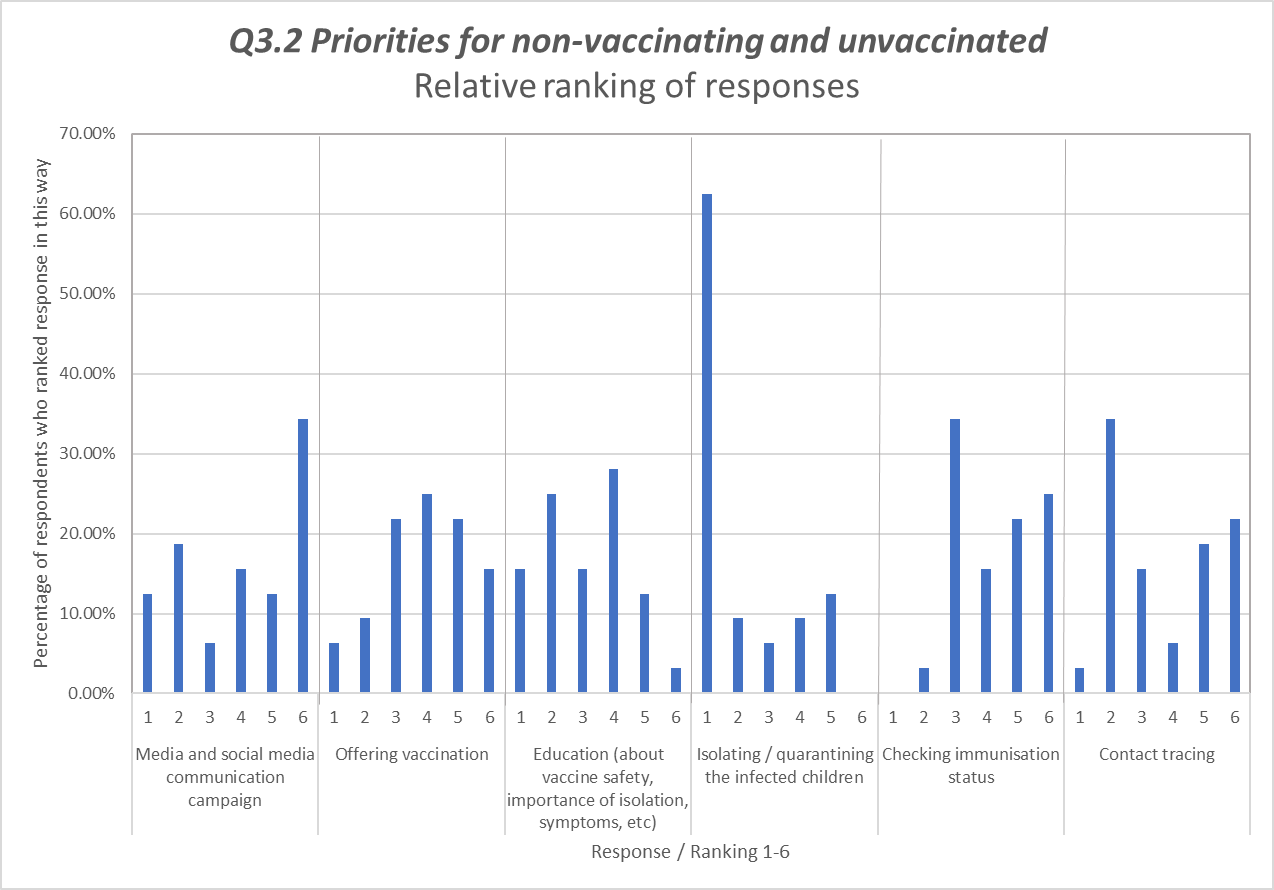


Additional File 3: Priorities for non-vaccinating and unvaccinated, relative rankings (Round 2)

Supplement: Supplementary file 3 — Additional file 3. Priorities for non-vaccinating and unvaccinated, relative rankings (Round 2). Graph showing relative ranking of responses. [file 12889_2021_10604_MOESM3_ESM.docx]

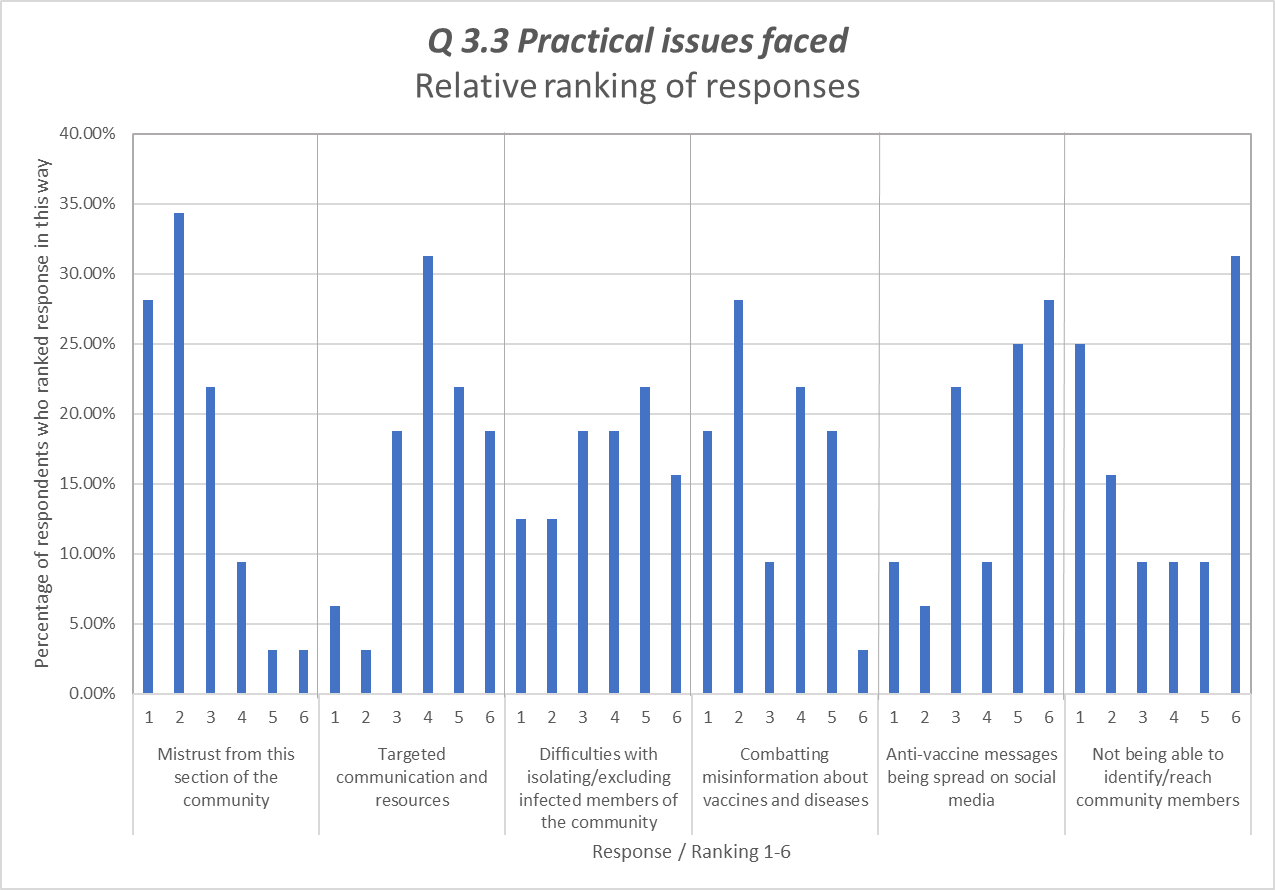


Additional File 4: Practical issues faced, Relative rankings (Round 2)

Supplement: Supplementary file 4 — Additional file 4. Practical issues faced, Relative rankings (Round 2). Graph showing relative ranking of responses. [file 12889_2021_10604_MOESM4_ESM.docx]
