# Supplementary material for: Public health practitioner perspectives on dealing with measles outbreaks if high anti-vaccination sentiment is present
Source: BMC Public Health. 2021 Apr 9;21:578. doi: 10.1186/s12889-021-10604-3 (PMC8032458; doi:10.1186/s12889-021-10604-3)
Supplement: Supplementary file 5 — Additional file 5. What panellists need from the non-vaccinating community, Relative rankings (Round 2). Graph showing relative ranking of responses. [file 12889_2021_10604_MOESM5_ESM.docx]

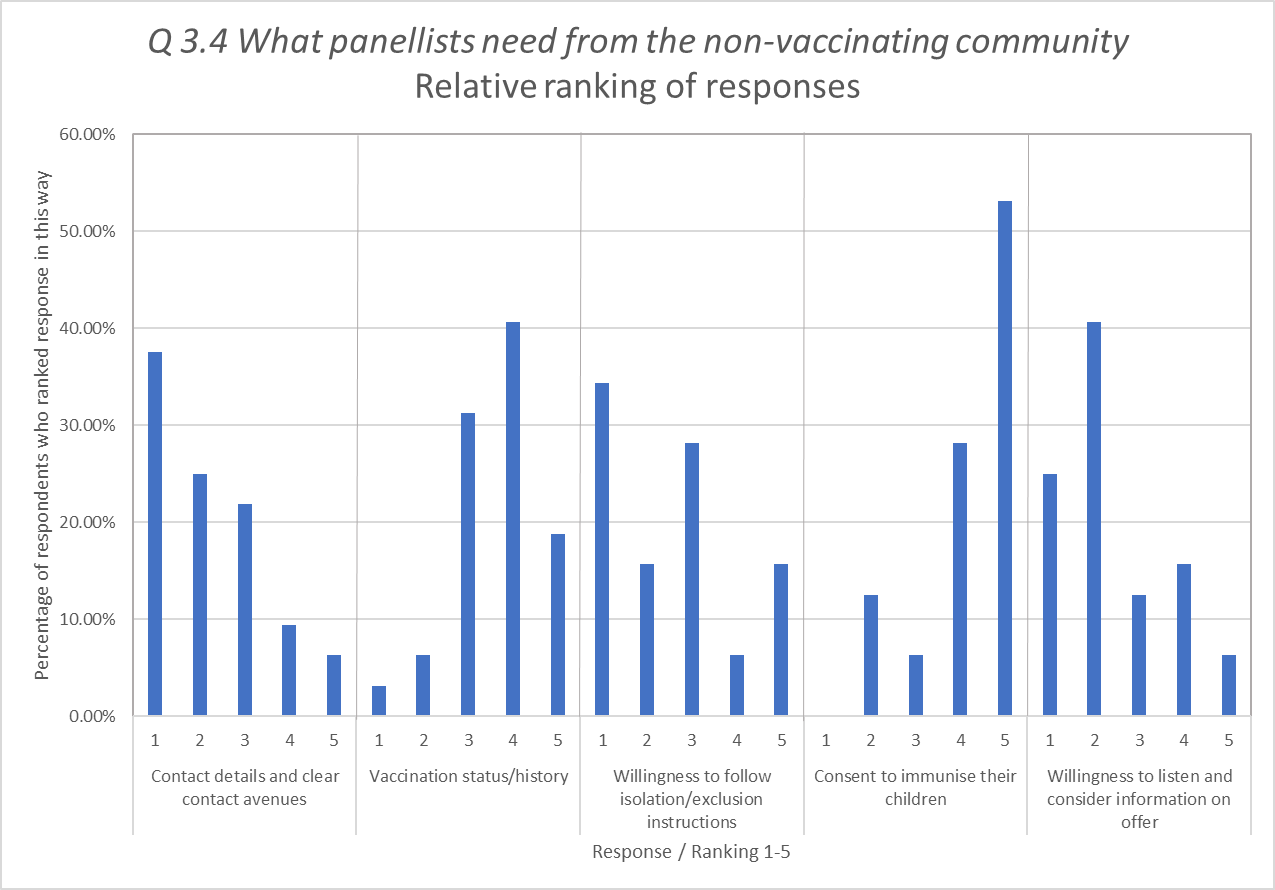


*Additional File 5: What panellists need from the non-vaccinating community, Relative rankings (Round 2)*
